# Ancient cell structural traits and photosynthesis in today’s environment

**DOI:** 10.1093/jxb/erx081

**Published:** 2017-04-28

**Authors:** José Javier Peguero-Pina, Domingo Sancho-Knapik, Eustaquio Gil-Pelegrín

**Affiliations:** 1 Unidad de Recursos Forestales, Centro de Investigación y Tecnología Agroalimentaria de Aragón, Gobierno de Aragón, Avda. Montañana 930, 50059, Zaragoza, Spain; 2 Instituto Agroalimentario de Aragón -IA2- (CITA-Universidad de Zaragoza), Zaragoza, Spain

**Keywords:** Cell wall elasticity, cell wall thickness, evolutionary constraints, gymnosperms, leaf anatomy, mesophyll conductance, photosynthesis, Rubisco, stomatal conductance

## Abstract

This article comments on:

Veromann-Jürgenson L-L, Tosens T, Laanisto L, Niinemets Ü. 2017. Extremely thick cell walls and low mesophyll conductance: welcome to the world of ancient living! Journal of Experimental Botany 68, 1639–1653.

**Mesophyll conductance to CO**_**2**_ **– a key factor in plant photosynthesis – is strongly influenced by leaf anatomy. In this issue, Veromann-Jürgenson *et al.* (pages 1639–1653) provide evidence of the conservation of ancient structural traits (extremely thick cell walls) in evolutionarily old taxa that suggest apparent evolutionary constraints on CO**_**2**_ **fixation. This opens the way for integrated approaches combining evolutionary constraints of diffusive, structural and biochemical factors on plant photosynthesis.**

For many decades, the rate of CO_2_ diffusion through stomata (stomatal conductance, *g*_s_) and the capacity of photosynthetic machinery to convert light to biochemical energy and fix CO_2_ into sugars (biochemical capacity) were considered the only two factors constraining plant photosynthesis. However, pioneer studies already suggested that CO_2_ diffusion from sub-stomatal cavities to carboxylation sites inside chloroplasts (mesophyll conductance, *g*_m_) could also limit photosynthesis (Nobel, 1970). There is now an increasing interest among plant physiologists in studying the role of *g*_m_ as the third major player involved in controlling the rate of photosynthesis, and this is reflected in the number of studies recently published addressing the ecophysiological significance of *g*_m_ and its regulatory mechanisms (see Flexas *et al.*, 2012, and references therein).

Large variations in *g*_m_ among species or plant groups can be explained through the existence of several barriers to CO_2_ diffusion across the mesophyll (including air, cell walls, lipid membranes, cytoplasm and chloroplast stroma) differing in nature and size (Evans *et al.*, 2009; Terashima *et al.*, 2011). Recently, a small number of studies have quantified the importance of different leaf anatomical traits in determining the variability in *g*_m_ and photosynthesis among species (Tomás *et al.*, 2013; Peguero-Pina *et al.*, 2016*a* ; Peguero-Pina *et al.*, 2017) or even within the same species growing under contrasting environmental conditions (Terashima *et al.*, 2011; Tosens *et al.*, 2012*a* ; Peguero-Pina *et al.*, 2016*b* , *c*). These analyses showed that *g*_m_ was most strongly correlated with the chloroplast surface area facing intercellular air spaces (*S*_c_/*S*), thickness of the mesophyll cell walls (*T*_cw_), and chloroplast size; however, depending on foliage structure, the overall importance of *g*_m_ in constraining photosynthesis and the importance of different anatomical traits in the restriction of CO_2_ diffusion varied (Evans *et al.*, 2009; Terashima *et al.*, 2011; Tosens *et al.*, 2012*b* ).

## Ancient structural traits constrain photosynthesis in old taxa

Mesophyll conductance has been estimated for more than 100 species from all major plant groups, but mainly spermatophytes (angiosperms and gymnosperms), with little data for ferns, liverworts and hornworts (Flexas *et al.*, 2012; Carriquí *et al.*, 2015; Tosens *et al.*, 2016). Considerable variations in *g*_m_ and its underlying traits among different plant groups have supported the hypothesis that an evolutionary trend exists towards higher *g*_m_ together with the diversification of embryophytes (Flexas *et al.*, 2012; Carriquí *et al.*, 2015). However, there is still a significant knowledge gap concerning phylogenetic/evolutionary trends in *g*_m_.

The number of studies concerning *g*_m_ in gymnosperms is surprisingly limited, in spite of the great importance of coniferous forests throughout the world (Breckle, 2002). Specifically, *g*_m_ had only been estimated in 13 conifer species before the study by Veromann-Jürgenson *et al.* (2017; see also references therein). Although gymnosperms show the lowest *g*_m_ values across spermatophytes (Flexas *et al.*, 2012), available data show a high degree of interspecific variation and suggest the primary role of *g*_m_ as a limiting factor for net CO_2_ assimilation in conifers. However, as pointed out by Veromann-Jürgenson *et al.* (2017), information about *g*_m_ with its underlying structural traits is especially limited in conifers, and only Peguero-Pina *et al.* (2012, 2016*b* ) had previously correlated *g*_m_ with ultrastructural needle anatomy in species belonging to this plant group.

In this context, Veromann-Jürgenson *et al.* (2017) have characterized the structural traits (i.e. *S*_c_/*S*, chloroplast size and *T*_cw_) that are mainly responsible for low *g*_m_ and photosynthetic performance in several evolutionarily old gymnosperms and herbaceous species with contrasting phylogenetic age. These authors have found, for the first time, striking evidence about the effect of divergence time on structure and physiology, and specifically a negative correlation between estimated evolutionary age of the plant genus and area-based photosynthesis (*A*_N_). However, as they recognize, this statement should be treated with caution because species’ evolutionary adaptation to prevailing environmental conditions can actually drive photosynthetic capacity more strongly than their evolutionary age (Tosens *et al.*, 2016). Regarding CO_2_ diffusion across the mesophyll, although *g*_m_ itself was not related to plant evolutionary age, the lowest *g*_m_ values (which scaled positively with *A*_N_ regardless of evolutionary age) were observed for the oldest genera.

The most significant conclusion emerging from the study of Veromann-Jürgenson *et al.* (2017) is that the preservation of old traits suggests constraints on evolution due to the co-occurrence of low *g*_m_ and *A*_N_ and the corresponding high *T*_cw_ for species with widely contrasting ecological strategies. Thereby, these authors hypothesize that (i) the high-CO_2_ atmosphere when several of these thick-cell-walled species evolved (about 65–200 million years ago) suggests a lower control of diffusional limitations on the rate of photosynthesis, and (ii) the preservation of this ancient trait in spite of the gradual CO_2_ decrease through evolution has led to stronger control of foliage assimilation rates by *g*_m_ (Box 1).

**Box 1. Mesophylls of evolutionarily old or modern species which have evolved under different CO_2_ concentrations**

The schematic representation shows the mesophyll of (A) an evolutionarily old species which evolved under high CO_2_ concentration and (B) an evolutionarily modern species which evolved under low CO_2_ concentration. Photosynthesis in evolutionarily old species at current CO_2_ concentrations could be constrained by low values of stomatal conductance (*g*_s_) (due to larger stomatal size but lower stomatal density: Franks and Beerling, 2009), low values of mesophyll conductance (*g*_m_) (due to extremely thick cell walls, *T*_cw_: Veromann-Jürgenson *et al.*, 2017), and lower carboxylase catalytic efficiency (*k*_cat_^c^/*K*_c_) (Galmés *et al.*, 2014).

**Figure:**
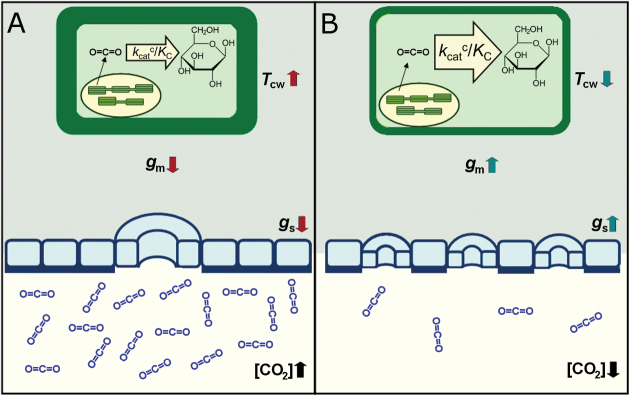

## Integrated approaches: the way forward

The phylogenetic trend consisting of a reduction of the cell wall thickness through evolution from bryophytes to angiosperms was recently considered by Carriquí *et al.* (2015), who suggested that this reduction was probably crucial to allow plants to achieve larger photosynthetic rates albeit at the expense of a reduction in desiccation tolerance. Increased values of cell wall thickness have been related to a greater ability to preserve the structure of the cells under water stress (Proctor and Tuba, 2002; Carriquí *et al.*, 2015). Related to this, Corcuera *et al.* (2002) suggested that cell wall thickness may be associated with the maximum bulk modulus of elasticity ( *ε*_max_), one of the main physiological traits related to the functional role of the cell wall. Higher *ε*_max_ values are seen as an efficient mechanism for plant performance under dry climates, as low cell-wall elasticity (i.e. high *ε*_max_) would allow a rapid recovery after a decrease in soil water content (Corcuera *et al.*, 2002). To the best of our knowledge, there are no published studies empirically relating higher *ε*_max_ values with increasing cell wall thickness. However, there does seem to be a positive trend between both parameters when values of cell wall thickness are plotted against *ε*_max_ for several oak species (Box 2). Additional studies including simultaneous measurements of both parameters in a larger number of species from different genera are required for understanding the ultimate causal factors involved in this trade-off.

**Box 2. Cell wall thickness and maximum bulk modulus of elasticity**

The graph shows the relationship between cell wall thickness (*T*_cw_) and the maximum bulk modulus of elasticity ( *ε*_max_) for several *Quercus* species. Mean values of *ε*_max_ are from Corcuera *et al.* (2002); mean values of cell wall thickness are from Peguero-Pina *et al.* (2016*a* , 2017).

**Figure:**
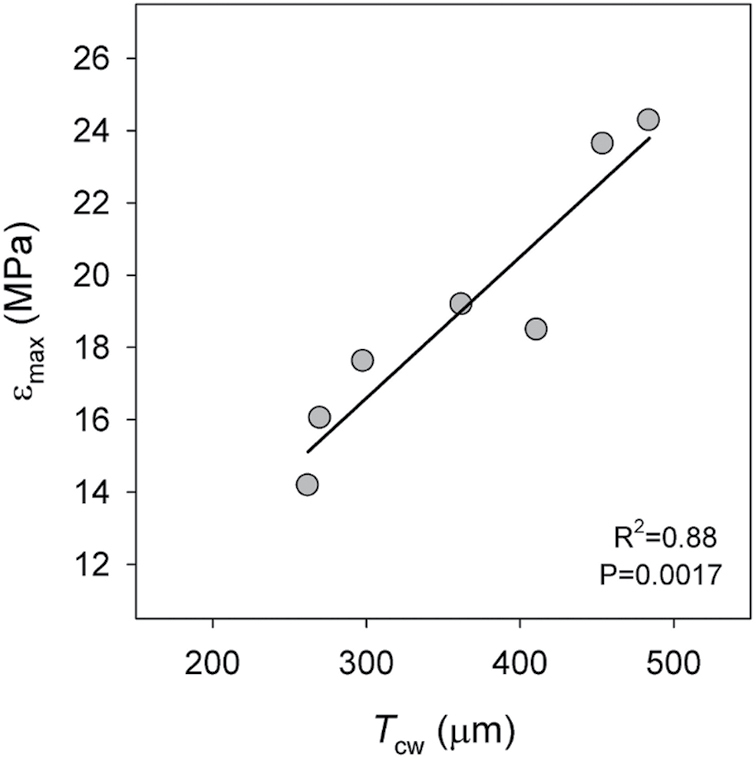

Besides *g*_m_, Veromann-Jürgenson *et al.* (2017) found that *A*_N_ also depended strongly on *g*_s_, which correlated negatively with the age of the genus. This empirical result is supported by Franks and Beerling (2009), who stated that periods of falling atmospheric CO_2_ challenged plants with diminished CO_2_ availability, inducing a selection for higher maximum *g*_s_ through a trend towards smaller stomatal size and higher density, thereby alleviating the negative impact of diminishing CO_2_ on photosynthesis (Box 1). This co-regulation between *g*_m_ and *g*_s_ is to some extent expected (Flexas *et al.*, 2012) because CO_2_ and water vapour share, in part, diffusion pathways in the mesophyll (Evans *et al.*, 2009; Terashima *et al.*, 2011).

Beyond diffusive components (i.e. *g*_s_ and *g*_m_), other factors also determine the rate of plant photosynthesis, such as the enzyme ribulose-1,5-bisphosphate carboxylase/oxygenase (Rubisco). Galmés *et al.* (2014) found evolutionary trends in relation to atmospheric CO_2_ when analyzing the variability in Rubisco kinetics in different plant species. These authors confirmed that evolution of Rubisco towards increased affinity for CO_2_ (*K*_c_ falling) and increased carboxylase catalytic efficiency (*k*_cat_^c^/*K*_c_) in land plants is likely to have been complementary to falling CO_2_/O_2_ ratios, as well as to adaptations in leaf architecture, morphology and conductance (Beerling *et al.*, 2001; Franks and Beerling, 2009; Haworth *et al.*, 2011) (Box 1).

Veromann-Jürgenson *et al.* (2017) provide an interesting starting point for further studies on the role of phylogenetic aspects in plant physiological performance (i.e. the influence of the age on photosynthesis associated with the preservation of ancient traits in evolution, such as extremely thick cell walls). Currently, the way forward is through the implementation of integrated approaches that combine evolutionary constraints of diffusive, structural and biochemical factors on plant photosynthetic performance, together with other functional traits (e.g. plant hydraulics).
